# Comparative Mitogenomic Analyses of Tanypodinae (Diptera: Chironomidae)

**DOI:** 10.3390/insects16020203

**Published:** 2025-02-12

**Authors:** Xiu-Ru Xiao, Meng-Han Chen, Shu-Yi Li, Bing-Xin Guo, Yan Zhang, Zhi-Chao Zhang, Ya-Jun Qiao, Xiao-Long Lin

**Affiliations:** 1 Engineering Research Center of Environmental DNA and Ecological Water Health Assessment, Shanghai Ocean University, Shanghai 201306, China; zyxr1023@hotmail.com (X.-R.X.); chen_menghan124@163.com (M.-H.C.); shuyilll@163.com (S.-Y.L.); 17856074939@163.com (Y.Z.); zzc514644@gmail.com (Z.-C.Z.); 2 Shanghai Universities Key Laboratory of Marine Animal Taxonomy and Evolution, Shanghai Ocean University, Shanghai 201306, China; 3 Science and Technology Innovation Bureau, Guangming District, Shenzhen 518000, China; aquagbx@163.com; 4 Ecology and Environment Bureau of Xiong’an New Area, Baoding 071000, China

**Keywords:** characteristics, mitogenome, phylogeny

## Abstract

Tanypodinae, a highly diverse subfamily within Chironomidae (Diptera), has been poorly represented in mitochondrial genome (mitogenome) databases, with only a limited number of complete or partial sequences available. To address this gap, we present the first complete mitogenome sequences of 16 Tanypodinae species and 1 Podonominae species. By integrating these novel data with previously published chironomid mitogenomes, we conducted a comprehensive comparative mitogenomic analysis of 21 Tanypodinae species. Our results reveal that Tanypodinae mitogenomes maintain a conserved structural organization, preserving the ancestral insect gene arrangement. The nucleotide composition exhibits significant bias, with the control region demonstrating the highest A + T content among all genomic regions. Evolutionary analysis indicates that all protein-coding genes (PCGs) are subject to purifying selection, with *ATP8* exhibiting the most rapid evolutionary rate. Phylogenetic reconstruction based on PCG amino acid sequences delineates seven well-supported clades within Tanypodinae, corresponding to the tribes Pentaneurini, Natarsiini, Procladiini, Tanypodini, Clinotanypodini, Macropelopiini, and Anatopyniini. These findings significantly advance our understanding of mitogenomic architecture and provide critical insights into the phylogenetic relationships within Tanypodinae.

## Simple Summary

This study aims to elucidate the fundamental characteristics of the mitochondrial genomes (mitogenomes) and phylogenetic relationships of Tanypodinae. We provide the first complete mitogenome of 16 Tanypodinae species and 1 Podonominae species. Through a comprehensive comparative genomic analysis of 21 Tanypodinae mitogenomes, including previously published data, we systematically investigated their mitogenomic features, including AT content, codon usage patterns, and selection pressure. Furthermore, we reconstructed the phylogenetic relationships within Tanypodinae using both maximum likelihood and Bayesian inference methods, integrating all available mitogenomic data. This study significantly expands the Chironomidae mitogenome database and provides novel insights into the evolutionary characteristics and phylogenetic framework of Tanypodinae.

## 1. Introduction

**Figure 1:**
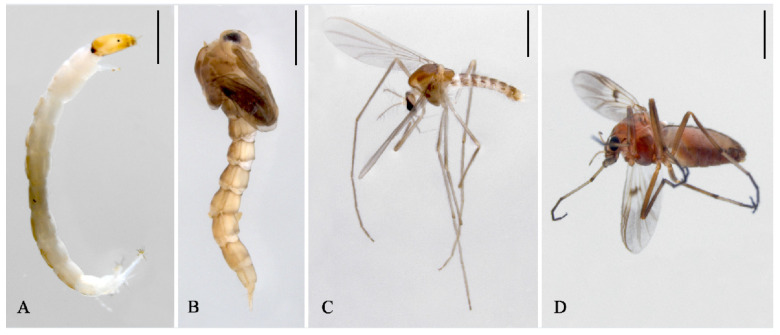
Photos of Tanypodinae: (**A**) larva of *Trissopelopia*, (**B**) pupa of *Trissopelopia*, (**C**) adult male of *Trissopelopia*, and (**D**) adult female of *Procladius longistilus*. Scale bars = 1 mm. Photos were photographed by Xiao-Long Lin, Shanghai Ocean University.

Chironomidae, a globally distributed aquatic dipteran family, occupies diverse habitats across all zoogeographic regions [1,2]. Within this family, Tanypodinae (Figure 1) represents the third most species-rich subfamily, exhibiting a near-cosmopolitan distribution, with the exception of Antarctica. This subfamily displays remarkable diversity in both morphological characteristics and ecological adaptations, yet its phylogenetic relationships remain inadequately understood and require comprehensive molecular investigation. Although traditional morphological approaches have provided substantial support for tribal classification at deeper taxonomic levels, certain phylogenetic relationships persist as unresolved [3]. The recent proliferation of molecular data within Chironomidae has significantly enhanced our understanding of their evolutionary history [4,5,6]. Contemporary morphological and molecular phylogenetic studies have consistently supported the monophyly of most Tanypodinae tribes [7,8]. Nevertheless, prior to the current investigation, the mitogenomic resources for this subfamily were remarkably limited, with only five Tanypodinae species having reported mitogenomes [9,10,11,12], thereby constraining our understanding of both mitochondrial architecture and phylogenetic patterns within this ecologically significant group.

To address these limitations and better understand the phylogenetic relationships of the tribes within Tanypodinae, we employed mitochondrial sequence analysis. The mitochondrial genome (mitogenome), characterized as a circular double-stranded DNA molecule spanning 14–20 kilobases in length [13,14], represents a fundamental component of eukaryotic cellular machinery. This compact genome typically comprises 37 genes, including 13 protein-coding genes (PCGs), 2 ribosomal RNA (rRNA) genes, 22 transfer RNA (tRNA) genes, and a functionally crucial control region (CR) responsible for regulatory functions [15,16]. Several distinctive features contribute to the mitogenome’s utility in evolutionary studies: its relatively short length, accelerated evolutionary rates compared to nuclear DNA, strict maternal inheritance pattern, and generally conserved gene order [17]. These unique characteristics have established the mitogenome as an invaluable molecular marker for investigating species evolution, population genetic structure, and phylogeographic patterns. Consequently, mitogenomic data have become extensively employed in taxonomic classification and phylogenetic reconstruction across diverse organisms [18,19]. In entomological research, the application of complete or partial mitogenome sequences has significantly advanced our understanding of insect systematics, population dynamics, and biogeographic history, providing critical insights into the evolutionary trajectories and adaptive mechanisms of this highly diverse taxonomic group [20,21,22]. Therefore, compared to genomics, mitogenomics offers advantages such as higher mutation rates, maternal inheritance, and cost-effectiveness, making it particularly valuable for evolutionary studies.

In this study, we present the first complete mitogenomes of 16 Tanypodinae species and 1 Podonominae species, significantly expanding the mitogenomic resources for Chironomidae. By integrating these novel data with publicly available Tanypodinae mitogenomes, we conducted a comprehensive phylogenetic analysis to explore the phylogenetic relationships among selected tanypod taxa.

## 2. Materials and Methods

### 2.1. Sampling, DNA Extraction, and Specimen Identification

Specimens representing 21 Tanypodinae species and 1 Podonominae species were collected from various locations across China (Table 1). Immediately after collection, all specimens were preserved in 85% ethanol and stored at 4 °C or below to maintain DNA integrity. Specimen identification of these specimens was performed through an integrated approach combining DNA barcoding analysis and detailed morphological examination [23,24,25,26,27]. Voucher specimens are stored at the College of Fisheries and Life Sciences, Shanghai Ocean University, Shanghai, China. Total genomic DNA was extracted from either thoracic tissue of adult specimens or larva skin using the Qiagen DNA blood and tissue kit (Qiagen, Hilden, Germany), following the manufacturer’s protocol.

### 2.2. Sequencing, Assembly, and Annotation

Whole-genome sequencing was performed for each species using the Illumina NovaSeq 6000 platform at Novogene Co., Ltd. (Beijing, China). Libraries were prepared with a 350 bp insert size and sequenced using a paired-end 150 strategy. Raw sequencing reads were quality-filtered and adapter-trimmed using Trimmomatic [29], generating over 2 Gb of high-quality clean data per sample. De novo assembly of mitogenomes was conducted using NOVOPlasty v4.3.1 (Brussel, Belgium) [30] with a k-mer value of 39, utilizing the cytochrome *c* oxidase I (*COI*) barcode sequence as the seed sequence. To validate the accuracy of the mitogenomes’ assembly, clean reads were mapped against the assembled sequences using Geneious v2024.0.5 (Boston, MA, USA) [31]. tRNA genes were identified and annotated using MITOS v2.1.7 (Greifswald, Germany) [32]. rRNA genes and PCGs were annotated through sequence aligning with homologous regions from *Clinotanypus yani* (MW373524) and *Tanypus punctipennis* (MZ475054) in Geneious. The complete mitogenome sequences of the newly sequenced species have been deposited in GenBank under accession numbers OP006225–OP006244.

### 2.3. Sequence Analysis and Substitution Rate

Nucleotide composition analysis, including the calculation of codon usage patterns and relative synonymous codon usage values, was performed using Geneious. Mitochondrial strand asymmetry was quantitatively assessed through the calculation of GC-skew and AT-skew indices, determined using the following formulas: GC-skew = [G − C]/[G + C] and AT-skew = [A − T]/[A + T]. To evaluate evolutionary selection pressures, synonymous substitution rates (Ks) and non-synonymous substitution rates (Ka) were computed using DnaSP v6.12.03 (Barcelona, Spain) [33], with subsequent calculation of the Ka/Ks ratio for each PCG.

### 2.4. Phylogenetic Analyses

A phylogenetic analysis was conducted using mitogenomes from 23 species, comprising 21 species of Tanypodinae as ingroups and 2 Podonominae species as the outgroups (Table 1). The 13 PCGs and two rRNAs from each species were individually aligned using Muscle (Carlsbad, CA, USA) [34], followed by refinement with trimAl v1.4.1 (Barcelona, Spain) [35] to optimize sequence alignment. Four datasets were constructed for phylogenetic analyses: (1) PCG123 (all codon positions of the 13 PCGs), (2) PCG12 (first and second codon positions of the 13 PCGs), (3) PCG123R (all codon positions of 13 PCGs plus two rRNAs), and (4) AA (amino acid sequences translated from the 13 PCGs) (Figure S1). Sequence concatenation for each dataset was performed using FASconCAT-G v1.04 (Santa Cruz, CA, USA) [36]. To ensure data quality, base substitution saturation was assessed for each gene and codon position using DAMBE v7.2.32 (Ottawa, ON, Canada) [37], confirming no evidence of saturation. Sequence divergence heterogeneity was evaluated using AliGROOVE v1.08 (Bonn, Germany) [38]. The optimal partitioning scheme and corresponding substitution models were determined using PartitionFinder 2.0 (Brisbane, Queensland, Australia) [39] under the Bayesian Information Criterion (Table S1). Phylogenetic reconstruction was performed using both maximum likelihood (ML) and Bayesian inference (BI) approaches. ML analysis was implemented in IQ-TREE v2.2.2.7 (Canberra, ACT, Australia) [40], with the best-fit substitution models and 1000 bootstrap replicates. BI analysis was conducted using MrBayes 3.2.7 (Uppsala, Sweden) [41], with two independent Markov Chain Monte Carlo (MCMC) runs of 10 million generations, sampling every 1000 generations, and discarding the first 25% of trees as burn-in. MCMC convergence was verified using Tracer 1.7 (Edinburgh, UK) [42]. To resolve inconsistencies among phylogenetic results from different datasets, we performed four-cluster likelihood mapping (FcLM) analysis using TREE-PUZZLE v5.3 (Munich, Germany) [43] to evaluate the robustness of the phylogenetic tree topology.

## 3. Results and Discussions

### 3.1. General Features of 21 Tanypodinae and 1 Podonominae Mitogenomes

We newly sequenced 19 species of Tanypodinae and 1 species of Podonominae, and, for the first time, report the complete mitogenomes of 16 Tanypodinae species and 1 Podonominae species. The complete mitogenomes of 21 Tanypodinae species were successfully sequenced, revealing lengths ranging from 15,782 bp (*Denopelopia bractea*) to 17,752 bp (*Thienemannimyia fuscipes*). The complete mitogenome of *Paraboreochlus okinawanus* was also determined, with a length of 17,647 bp. All mitogenomes contain the typical 37 genes, with the majority encoded on the heavy (H) strand, while 4 PCGs, 9 tRNAs, and 2 rRNAs are located on the light (L) strand. Comparative analysis reveals a strong AT bias across all Tanypodinae mitogenomes, with species-specific A + T content ranging from 74.44% (*T. fuscipes*) to 79.63% (*Larsia myagsensis*). Within the PCGs, the A + T content varies between 71.30% (*Saetheromyia tedoriprima*) and 76.43% (*T. punctipennis*). Notably, the third codon positions of PCGs display significantly higher A + T content compared to the first and second positions, which may reflect evolutionary adaptations to environmental pressures or translational efficiency optimization. All three codon positions exhibit negative AT-skew values, while GC-skew patterns vary across codon positions: positive at the first codon position but negative at the second and third codon positions. Additionally, the complete mitogenomes of Tanypodinae showed consistent positive AT-skew (0.01 to 0.05) and negative GC-skew (−0.13 to −0.21) values (Table S2).

### 3.2. Protein-Coding Genes

The PCGs in the mitogenomes exhibit length variation ranging from 165 bp (*ATP8*) to 1734 bp (*ND5*), with the combined length of all 13 PCGs spanning 11,209 to 11,224 bp, representing 63.19% to 71.02% of the total mitogenome length. Most of the PCGs start with the typical codon of ATN, while the initiation codon of partial *ND1* and *ND5* is TTG (18/21) and GTG (17/21), respectively (Table S3). The *COI* gene displays particularly diverse initiation patterns, with TCG predominating (15/21), followed by ACG (3/21) and TTG (3/21). For termination codons, TAA or TAG is the stop codon for most PCGs, with TAA being the most commonly used stop codon. However, the stop codon of the *COII* gene in all Tanypodinae species is a single T. Analysis of nucleotide composition reveals consistent trends in GC-skew and AT-skew values across PCGs, with positive GC-skew and negative AT-skew values indicating a higher abundance of T and G bases compared to A and C. This bias is particularly pronounced in the second and third codon positions, where T content reaches up to 51.17%, demonstrating significant codon position-specific nucleotide composition bias in the PCGs.

**Figure 2:**
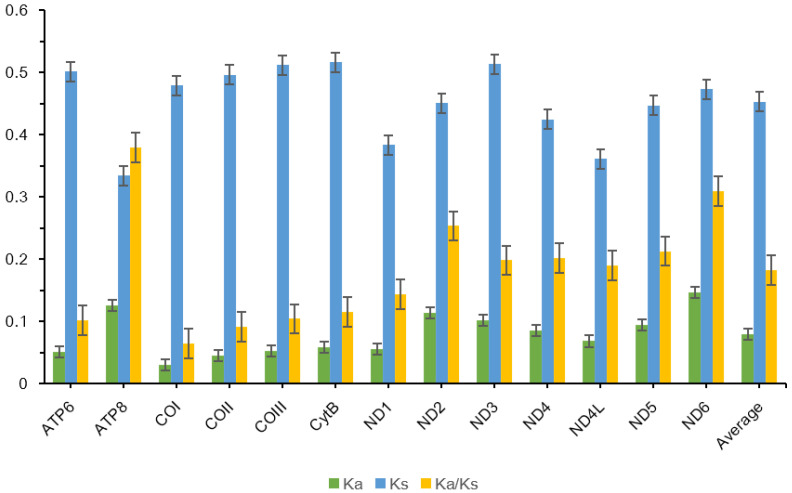
Evolutionary rate of the 13 PCGs of mitogenomes of 21 Tanypodinae species. Ka refers to non-synonymous nucleotide substitutions, Ks refers to synonymous nucleotide substitutions, and Ka/Ks refers to the evolution ratio of each PCG. The abscissa represents the 13 PCGs, and the ordinate represents Ka/Ks values.

The Ka/Ks ratio, a critical indicator of evolutionary selection pressure on PCGs, reveals distinct patterns across the mitogenome (Figure 2). The average Ka/Ks ratio across the 13 PCGs is 0.18, with individual gene values ranging from 0.07 (*COI*) to 0.38 (*ATP8*). All PCGs exhibit Ka/Ks ratios significantly below 1, providing strong evidence of pervasive purifying selection [44]. This evolutionary mechanism serves to eliminate deleterious mutations and maintain population stability through successive generations [45]. Notably, *ATP8* displays the highest Ka/Ks ratios among all PCGs, a pattern consistent with observations in other chironomid species. This elevated evolutionary rate may be attributed to *ATP8*’s heightened vulnerability to free radical attacks within the mitochondrial genome, subjecting it to intense selective pressure [46]. In contrast, *COI* demonstrates the lowest Ka/Ks ratio (0.07), reflecting its exceptionally conserved nature and critical functional role. Comprehensive analysis of Ka/Ks ratios coupled with heterogeneity assessment confirms that *ATP8* evolves at the most rapid rate among mitochondrial genes, while *COI* represents the most evolutionarily constrained gene in the Tanypodinae mitogenomes.

### 3.3. Mitochondrial Gene Codon Usage

**Figure 3:**
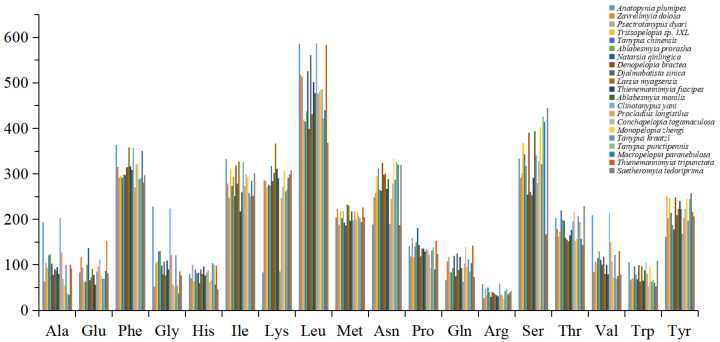
Amino acid distribution of mitogenomes of 21 Tanypodinae species. The X-axis represents the codon families, and the Y-axis represents the total codons.

Codon usage analysis reveals distinct patterns in amino acid encoding across the Tanypodinae mitogenomes. Eight amino acids—Ala, Gly, Leu1, Pro, Ser1, Ser2, Thr, and Val—are each encoded by four distinct codons, while the remaining amino acids are specified by two codons each. Comparative analysis of the 21 Tanypodinae species identifies Asn, Ile, Leu2, Lys, Phe, and Ser as the most frequently utilized amino acids, whereas Arg shows the lowest occurrence frequencies (Figure 3). This codon usage bias appears to be influenced by strand-specific base composition asymmetry, a phenomenon commonly observed in mitogenomes. The third codon positions exhibit a pronounced preference for A and T bases over G and C, resulting in the predominance of NNA and NNT codons across most mitochondrial genes. This pattern suggests that synonymous codon usage in Tanypodinae mitogenomes is strongly influenced by the underlying AT bias characteristic of mitochondrial DNA.

### 3.4. Transfer and Ribosomal RNA Genes

The lengths of the *12S rRNA* gene range from 801 bp (*T. fuscipes*) to 821 bp (*Trissopelopia* sp. 1XL), while the lengths of the *16S rRNA* gene range from 1372 bp (*D. bractea*) to 1428 bp (*S. tedoriprima*). The GC content of 12S ranges from 16.13% (*L. myagsensis*) to 21.62% (*Tanypus kraatzi*), while that of 16S ranges from 14.15% (*L. myagsensis*) to 19.05% (*T. punctipennis*). A total of 22 tRNA genes are identified in the mitogenomes of the 21 Tanypodinae species, with sizes ranging from 64 to 72 bp. The distribution of nucleotides in tRNA genes differs from that in rRNA genes in the mitogenome. Specifically, the rRNA genes display a negative GC-skew value, while the 22 tRNA genes exhibit a negative GC-skew value, which ranges from −0.12 to −0.07 across Tanypodinae species. The genetic code translation system maintained a typical one-to-one correspondence between codons and their respective anticodons, ensuring accurate protein synthesis within the mitochondrial translation machinery.

### 3.5. Heterogeneity Analysis

**Figure 4:**
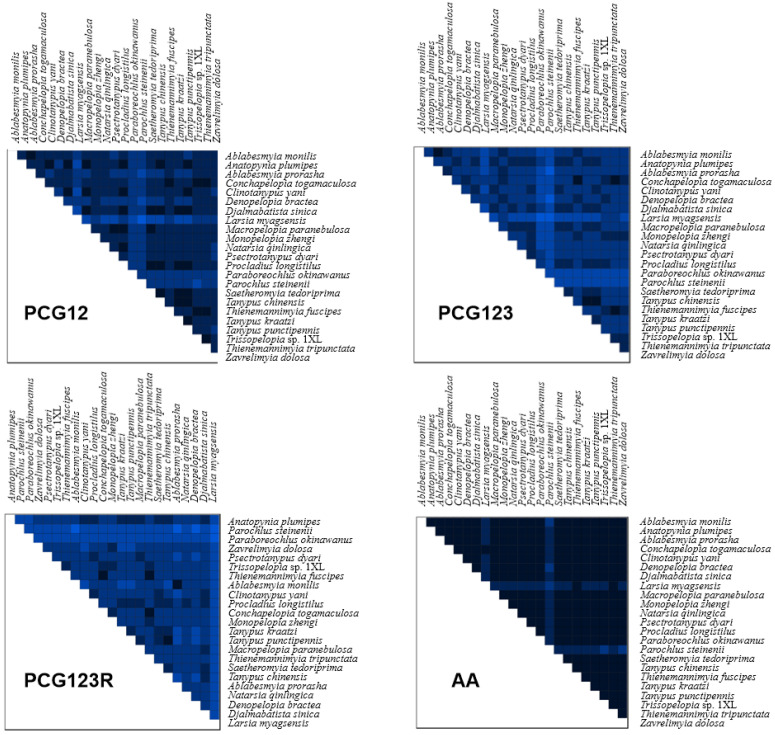
Heterogeneity analysis based on 13 PCGs and two rRNA sequences. The extent of sequence similarity is represented by colored blocks based on AliGROOVE scores ranging from −1 (strong heterogeneity between datasets; the color is red) to +1 (weak heterogeneity between datasets; the color is blue); the lighter the color of the colored block of each dataset, the stronger the heterogeneity, and the darker the color, the weaker the heterogeneity.

Sequence heterogeneity analysis provides valuable insights into the evolutionary divergence among Tanypodinae mitogenomes. Comparative assessment reveals distinct heterogeneity patterns across different datasets. The PCG123, PCG12, and AA datasets exhibit relatively low sequence heterogeneity in most pairwise comparisons, while the PCG123R dataset shows significantly higher heterogeneity levels (Figure 4). Notably, *L. myagsensis* shows significantly high sequence heterogeneity across all datasets, a phenomenon potentially attributable to either an accelerated evolutionary rate in this species or limited phylogenetic resolution resulting from insufficient sampling of Pentaneurini representatives [47]. Furthermore, the observed lower heterogeneity in PCG12 compared to PCG123 suggests an elevated evolutionary rate at the third codon position of PCGs. The AA dataset displays the lowest heterogeneity among all analyzed datasets, reflecting the conservative nature of amino acid sequences due to the degeneracy of the genetic code and the presence of synonymous codons.

### 3.6. Phylogenetic Analysis

**Figure 5:**
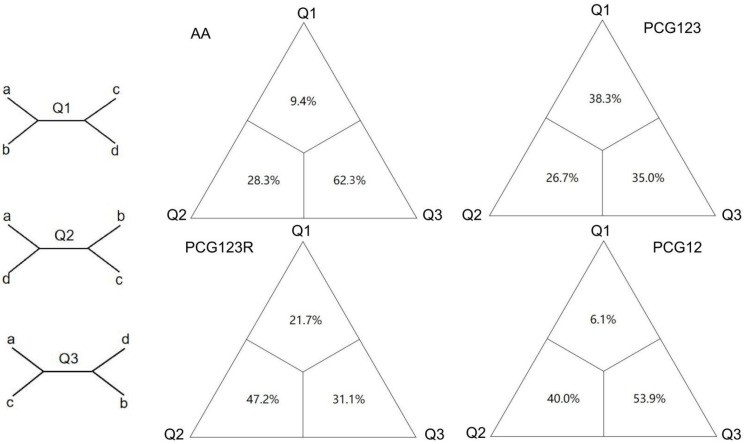
Four-cluster likelihood mapping (FcLM) of major clades of Tanypodinae. A priori groups in the analysis were a, outgroups; b, Nartarsiini clade; c, Pentaneurini clade; and d, remaining Tanypodinae clade: Macropelopiini, Tanpodini, Procladiini, Clinotanypodini, and Anatopyniini.

The observed discrepancies in phylogenetic reconstruction between amino acid and nucleotide sequence analyses primarily manifest in the topological positioning of the Natarsiini clade, thereby significantly impacting the inferred phylogenetic relationships among Tanypodinae tribes. As illustrated in Figure 5, the FcLM results derived from the AA dataset provide robust statistical support for a sister group relationship between Natarsiini and the clade comprising (Anatopyniini + (Macropelopiini + (Clinotanypodini + (Procladiini + Tanypodini)))). Based on these findings, we adopted the phylogenetic hypothesis generated from the AA dataset as the most reliable representation of Tanypodinae phylogenetic relationships, thereby further validating the utility and reliability of amino acid sequences in phylogenetic reconstruction.

**Figure 6:**
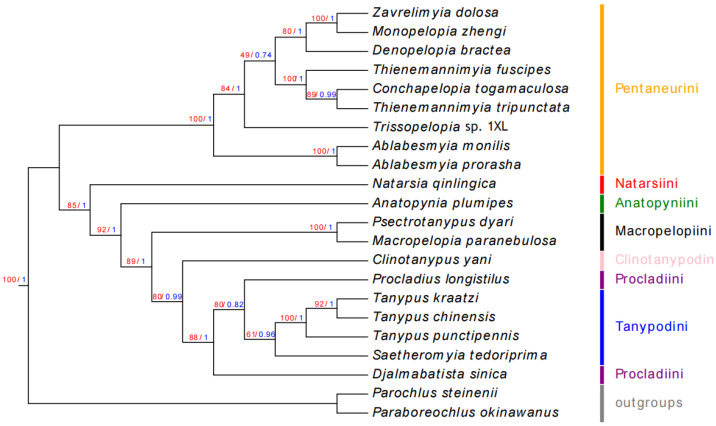
Phylogenetic trees of Tanypodinae inferred from the AA dataset (without *L. myagsensis*). Maximum likelihood tree for dataset AA bootstrap, with corresponding Bayesian posterior probabilities. The support of the two analyses was noted at the nodes. The values on the left and right are ML bootstrap values and BI posterior probabilities, respectively.

Phylogenetic analysis of the AA dataset using ML consistently supports the monophyly of Pentaneurini, irrespective of the inclusion of *L. myagsensis*, establishing this tribe as a sister group to other Tanypodinae lineages (Figure 6 and Figure S2–S8). Our investigation specifically targeted the monophyly of seven Tanypodinae tribes, Pentaneurini, Natarsiini, Procladiini, Tanypodini, Clinotanypodini, Macropelopiini, and Anatopyniini, with particular emphasis on resolving the internal relationships of Natarsiini. While the overall tree topology remained largely consistent across analyses, notable variations emerged at specific taxonomic levels. ML trees generally exhibit higher nodal support values compared to BI trees, with both methods demonstrating sensitivity to the influence of “rogue taxa” that exhibit positional instability across analyses. The monophyly of all seven tribes is consistently recovered, with the exception of Natarsiini, which displays variable positioning. The ML analysis of the AA dataset strongly supports the hypothesis that Pentaneurini constitutes a distinct clade that forms a sister group to the remaining Tanypodinae lineages.

The phylogenetic reconstruction delineates the 21 Tanypodinae species into seven distinct clades corresponding to the recognized tribes: Pentaneurini, Natarsiini, Procladiini, Tanypodini, Clinotanypodini, Macropelopiini, and Anatopyniini. The supported topology, represented as (Pentaneurini + (Natarsiini + (Anatopyniini + (Macropelopiini + (Clinotanypodini + (Procladiini + Tanypodini)))))), presents a novel perspective on inter-tribal relationships within Tanypodinae, and differs slightly from previous phylogenetic hypotheses [7]. Within Tanypodini, four species form a well-supported clade with the following structure: (((*Tanypus chinensis* + *T. kraatzi*) + *T. punctipennis*) + *S. tedoriprima*). Procladiini is represented by *P. longistilus* and *D. sinica*, which form a sister group to Tanypodini. The monophyletic Clinotanypodini clade, represented by *C. yani*, emerges as sister to (Procladiini + Tanypodini). Macropelopiini, comprising *Macropelopia paranebulosa* and *Psectrotanypus dyari*, forms a sister group relationship with (Clinotanypodini + (Procladiini + Tanypodini)). Natarsiini, represented by *Natarsia qinlingica*, clusters with the composite clade of (Anatopyniini + (Macropelopiini + (Clinotanypodini + (Procladiini + Tanypodini)))), forming a sister group to Pentaneurini. However, some nodes within Pentaneurini exhibit relatively low support values, potentially reflecting both the tribe’s extensive species diversity and insufficient sampling density. Notably, our analysis reveals that *Conchapelopia togamaculosa* is nested within the *Thienemannimyia* clade, suggesting potential taxonomic implications that warrant further investigation.

Insufficient taxon sampling limits the available data for other Tanypodinae, limiting a high-resolution analysis of the phylogenetic relationships. However, the phylogenetic relationships among Tanypodinae are not well supported at the species level, likely due to the relatively rapid mutation rates observed in the mitogenomes of most Tanypodinae species. A notable example is the extended branch length observed in *L. myagsensis*, which likely reflects elevated mutation rates in its rRNA sequences compared to other species. Despite significant advancements in molecular phylogenetic methodologies applied to Tanypodinae in recent years [48], persistent challenges remain in resolving deep-level tribal relationships. Our findings present a novel phylogenetic hypothesis regarding Natarsiini, strongly supporting its monophyletic status and sister group relationship with the clade comprising (Anatopyniini + (Macropelopiini + (Clinotanypodini + (Procladiini + Tanypodini)))). This discovery provides valuable insights into the evolutionary dynamics of Tanypodinae while highlighting the need for expanded taxonomic sampling to further refine our understanding of this diverse subfamily.

## 4. Conclusions

In this study, we present the first complete mitogenomes of 16 Tanypodinae species and 1 Podonominae species, significantly expanding the mitogenomic resources for chironomids. We characterized the mitogenomes of available Tanypodinae. Phylogenetic reconstruction based on mitogenomes robustly supports the monophyly of seven distinct clades within Tanypodinae, with the following phylogenetic relationships: (Pentaneurini + (Natarsiini + (Anatopyniini + (Macropelopiini + (Clinotanypodini + (Procladiini + Tanypodini)))))). Our findings highlight the critical need for expanded sampling within Pentaneurini, as increased taxonomic representation would substantially enhance our understanding of phylogenetic relationships and provide greater resolution to the phylogenetic structure of this diverse subfamily.

## Figures and Tables

**Table 1:** Taxonomic information, collection metadata, GenBank accession numbers, and references of mitogenomes used in the study.

Subfamily	Species	Sample ID	Life Stage	Sampling Metadata	GenBank Accession	Reference
Tanypodinae	*Ablabesmyia monilis*	XL1622	Adult male	Antu, Yanbian, Jilin, China, 42.457° N, 128.144° E, 12 July 2016, leg. C. Song.	OP006242	this study
Tanypodinae	*Ablabesmyia prorasha*	sun319	Adult male	Baisha, Hainan, China, 19.116° N, 109.092° E, leg. Y. Fu.	OP006228	this study
Tanypodinae	*Anatopynia plumipes*	CHLA111	Larva	Zhalong wetland, Heilongjiang, China, 47.168° N, 124.172° E, 1 October 2019, leg. M.-H. Liu	OP006225	this study
Tanypodinae	*Clinotanypus yani*	XL2042	Adult male	Jiulongshan Nature Reserve, Guangyuan, Sichuan, China, 31.976° N, 106.036° E, 8 August 2017, leg. C. Song	MW373524	[9]
Tanypodinae	*Conchapelopia togamaculosa*	XL2451	Adult male	Xuanen, Enshi, Hubei, China, 29.669° N, 109.609° E, 11 July 2015, leg. B.-J. Sun	OP006233	this study
Tanypodinae	*Denopelopia bractea*	sun359	Adult male	Kaihua, Quzhou, Zhejiang, China, 29.177° N, 118.120° E, 17 April 2011, leg. X.-L. Lin	OP006240	this study
Tanypodinae	*Djalmabatista sinica*	sun456	Adult male	Changjiang, Hainan, China, 19.116° N, 109.092° E, 31 March 2016, leg. B.-J. Sun	OP006241	this study
Tanypodinae	*Larsia myagsensis*	sun465	Adult male	Yinggeling, Baisha, Hainan, China, 19.083° N, 109.500° E, 14 March 2016, leg. B.-J. Sun	OP006230	this study
Tanypodinae	*Macropelopia* *paranebulosa*	XL2893	Larva	Benxi, Liaoning, China, 41.289° N, 124.898° E, 3 September 2014, leg. C. Song	OP006236	this study
Tanypodinae	*Monopelopia zhengi*	XL2560	Adult male	Ledong, Hainan, China, 18.693° N, 108.796° E, 8 March 2016, leg. B.-J. Sun	OP006234	this study
Tanypodinae	*Natarsia* *qinlingica*	sun342	Adult male	Yinzhou, Ningbo, Zhejiang, China, 29.804° N, 121.788° E, 10 May 2012, leg. X.-L. Lin	OP006229	this study
Tanypodinae	*Psectrotanypus dyari*	NLCH409	Larva	Shixing, Shaoguan, Guangdong, China, 24.723° N, 114.257° E, 24 August 2020, leg. X.-L. Lin	OP006244	this study
Tanypodinae	*Procladius longistilus*	XL2158	Adule female	Luotian, Huanggang, Hubei, China, 31.099° N, 115.734° E, 7 July 2019, leg. S. Qiu	OP006232	this study
Tanypodinae	*Saetheromyia tedoriprima*	XL3064	Adult male	Menghai, Xishuangbanna, Yunnan, China, 22.055° N, 99.990° E, 6 May 2013, leg. X.-L. Lin	OP006243	this study
Tanypodinae	*Tanypus* *chinensis*	sun245	Adult male	Deqing, Huzhou, Zhejiang, China, 28.980° N, 118.959° E, leg. 15 July 2012, leg. X.-L. Lin	OP006227	this study
Tanypodinae	*Tanypus kraatzi*	XL2603	Larva	Baoding, Hebei, China, 38.321° N, 115.375° E, 9 May 2018, leg. X.-L. Lin	OP006235	this study
Tanypodinae	*Tanypus punctipennis*	XL2604	Larva	Baoding, Hebei, China, 38.320° N, 115.375° E, 9 May 2018, leg. X.-L. Lin	MZ475054	[12]
Tanypodinae	*Thienemannimyia fuscipes*	XL1540	Adult male	Wuying, Yichun, Heilongjiang, China, 48.087° N, 129.247° E, 27 July 2016, leg. C. Song	OP006231	this study
Tanypodinae	*Thienemannimyia tripunctata*	XL3034	Adult male	Mengla, Xishuangbanna, Yunnan, China, 21.928° N, 101.255° E, 22 April 2014, leg. Q. Wang	OP006237	this study
Tanypodinae	*Trissopelopia* sp. 1XL	NLCH909	Larva	Long, Guilin, Guangxi, China, 25.625° N, 109.914° E, 16 August 2020, leg. Y. Yao	OP006226	this study
Tanypodinae	*Zavrelimyia dolosa*	LJHZ33	Adult male	Hechi, Guangxi, China, 24.731° N, 107.896° E, 5 June 2020, leg. Z.-N. Yang	OP006239	this study
Podonominae	*Paraboreochlus okinawanus*	LGS290	Adult male	Leishan, Qiandongnan, Guizhou, China, 26.396° N, 108.260° E, 18 January 2021, leg. H.-J. Yu	OP006238	this study
Podonominae	*Parochlus steinenii*	KT003702		King George Island, West, Antarctica, 62.233° S, 58.783° W, summer in 2015	KT003702	[28]

## Data Availability

The following information was supplied regarding the availability of DNA sequences: the new mitogenomes are deposited in GenBank of NCBI under accession numbers OP006225–OP006244.

## Supplementary Materials

The following supporting information can be downloaded at https://www.mdpi.com/article/10.3390/insects16020203/s1: Table S1. The best model for each partition of the four datasets. Table S2. Nucleotide composition of mitochondrial genomes of 21 Tanypodinae species. Table S3. Start and stop codons of 13 protein-coding genes in the mitogenomes of 21 Tanypodinae species. Figure S1. Substitution patterns of the PCG12 (a), PCG123 (b), and PCG123R (c) datasets. The graphs represent the increase in GTR distance. Figure S2. Phylogenetic trees of Tanypodinae inferred from the AA dataset (with *Larsia myagsensis*). (a) Bayesian inference tree. Numbers at the nodes are BI posterior probabilities. (b) Maximum likelihood tree. Numbers at the nodes are ML bootstrap values. Figure S3. Phylogenetic trees of Tanypodinae inferred from the PCG123 dataset (with *L. myagsensis*). (a) Bayesian inference tree. Numbers at the nodes are BI posterior probabilities. (b) Maximum likelihood tree. Numbers at the nodes are ML bootstrap values. Figure S4. Phylogenetic trees of Tanypodinae inferred from the PCG123R dataset (with *L. myagsensis*). (a) Bayesian inference tree. Numbers at the nodes are BI posterior probabilities. (b) Maximum likelihood tree. Numbers at the nodes are ML bootstrap values. Figure S5. Phylogenetic trees of Tanypodinae inferred from the PCG12 dataset (with *L. myagsensis*). (a) Bayesian inference tree. Numbers at the nodes are BI posterior probabilities. (b) Maximum likelihood tree. Numbers at the nodes are ML bootstrap values. Figure S6. Phylogenetic trees of Tanypodinae inferred from the PCG123 dataset (without *L. myagsensis*). (a) Bayesian inference tree. Numbers at the nodes are BI posterior probabilities. (b) Maximum likelihood tree. Numbers at the nodes are ML bootstrap values. Figure S7. Phylogenetic trees of Tanypodinae inferred from the PCG123R dataset (without *L. myagsensis*). (a) Bayesian inference tree. Numbers at the nodes are BI posterior probabilities. (b) Maximum likelihood tree. Numbers at the nodes are ML bootstrap values. Figure S8. Phylogenetic trees of Tanypodinae inferred from the PCG12 dataset (without *L. myagsensis*). (a) Bayesian inference tree. Numbers at the nodes are BI posterior probabilities. (b) Maximum likelihood tree. Numbers at the nodes are ML bootstrap values.

## References

[1] Li X.-Y., Yan L.-P., Pape T., Gao Y.-Y., Zhang D. (2020). Evolutionary insights into bot flies (Insecta: Diptera: Oestridae) from comparative analysis of the mitochondrial genomes. Int. J. Biol. Macromol..

[2] Li S.-Y., Zhao Y.-M., Guo B.-X., Li C.-H., Sun B.-J., Lin X.-L. (2022). Comparative analysis of mitogenomes of *Chironomus* (Diptera: Chironomidae). Insects.

[3] Silva F.L.D., Ekrem T. (2016). Phylogenetic relationships of nonbiting midges in the subfamily Tanypodinae (Diptera: Chironomidae) inferred from morphology. Syst. Entomol..

[4] Zheng C.-G., Liu Z., Zhao Y.-M., Wang Y., Bu W.-J., Wang X.-H., Lin X.-L. (2022). First report on mitochondrial gene rearrangement in non-biting midges, revealing a synapomorphy in *Stenochironomus* Kieffer (Diptera: Chironomidae). Insects.

[5] Zhang D., He F.-X., Li X.-B., Aishan Z., Lin X.-L. (2023). New mitogenomes of the Polypedilum generic complex (Diptera: Chironomidae): Characterization and phylogenetic implications. Insects.

[6] Allegrucci G., Carchini G., Todisco V., Convey P., Sbordoni V. (2006). A molecular phylogeny of Antarctic Chironomidae and its implications for biogeographical history. Polar Biol..

[7] Cranston P.S., Hardy N.B., Morse G.E. (2012). A dated molecular phylogeny for the Chironomidae (Diptera). Syst. Entomol..

[8] Siri A., Donato M. (2015). Phylogenetic analysis of the tribe Macropelopiini (Chironomidae: Tanypodinae): Adjusting homoplasies. Zool. J. Linn. Soc..

[9] Zheng C.-G., Zhu X.-X., Yan L.-P., Yao Y., Bu W.-J., Wang X.-H., Lin X.-L. (2021). First complete mitogenomes of Diamesinae, Orthocladiinae, Prodiamesinae, Tanypodinae (Diptera: Chironomidae) and their implication in phylogenetics. PeerJ.

[10] Lei T., Zheng X., Song C., Jin H., Chen L., Qi X. (2024). Limited Variation in Codon Usage across Mitochondrial Genomes of Non-Biting Midges (Diptera: Chironomidae). Insects.

[11] Gao S., Wang C., Tang Y., Zhang Y., Ge X., Zhang J., Liu W. (2024). Complete Mitochondrial Genome of *Tanypus chinensis* and *Tanypus kraatzi* (Diptera: Chironomidae): Characterization and Phylogenetic Implications. Genes.

[12] Jiang Y.-W., Zhao Y.-M., Lin X.-L. (2022). First report of the complete mitogenome of *Tanypus punctipennis* Meigen, 1818 (Diptera, Chironomidae) from Hebei Province, China. Mitochondrial DNA B.

[13] Boore J.L. (1999). Animal mitochondrial genomes. Nucleic Acids Res..

[14] Manchekar M., Scissum-Gunn K., Song D., Khazi F., Mclean S.L., Nielsen B.L. (2006). DNA recombination activity in soybean mitochondria. J. Mol. Biol..

[15] Song N., Li X., Yin X., Li X., Xi Y. (2020). The mitochondrial genomes of ladybird beetles and implications for evolution and phylogeny. Int. J. Biol. Macromol..

[16] Ge X., Wang J., Zang H., Chai L., Liu W., Zhang J., Yan C., Wang B. (2024). Mitogenomics Provide New Phylogenetic Insights of the Family Apataniidae (Trichoptera: Integripalpia). Insects.

[17] Ge X., Peng L., Vogler A.P., Morse J.C., Yang L., Sun C., Wang B. (2023). Massive gene rearrangements of mitochondrial genomes and implications for the phylogeny of Trichoptera (Insecta). Syst. Entomol..

[18] Saccone C., De Giorgi C., Gissi C., Pesole G., Reyes A. (1999). Evolutionary genomics in Metazoa: The mitochondrial DNA as a model system. Gene.

[19] Wolstenholme D.R. (1992). Animal mitochondrial DNA: Structure and evolution. Int. Rev. Cytol..

[20] Bian D., Dai M., Ye W., Lu Z., Li M., Fang Y., Qu J., Su W., Li F., Sun H. (2020). Complete mitochondrial genome of Spilosoma lubricipedum (Noctuoidea: Erebidae) and implications for phylogeny of noctuid insects. Genomics.

[21] Hao X., Liu J., Chiba H., Xiao J., Yuan X. (2021). Complete mitochondrial genomes of three skippers in the tribe Aeromachini (Lepidoptera: Hesperiidae: Hesperiinae) and their phylogenetic implications. Ecol. Evol..

[22] Ge X., Peng L., Morse J.C., Wang J., Zang H., Yang L., Sun C., Wang B. (2024). Phylogenomics resolves a 100-year-old debate regarding the evolutionary history of caddisflies (Insecta: Trichoptera). Mol. Phylogenet. Evol..

[23] Cheng M., Wang X. (2005). *Denopelopia* Roback & Rutter from China with emendation of the generic diagnosis (Diptera: Chironomidae: Tanypodinae). Zootaxa.

[24] Cheng M., Wang X.H. (2006). *Natarsia* Fittkau (Diptera: Chironomidae: Tanypodinae) from China. Zootaxa.

[25] Cheng M., Wang X. (2008). New species of *Clinotanypus* Kieffer, 1913 (Chironomidae: Tanypodinae) from China. Zootaxa.

[26] Duan X., Chang T., Jiao K.-L., Wang X.-H., Lin X.-L. (2021). *Monopelopia* Fittkau, 1962, a newly recorded genus from Oriental China (Diptera, Chironomidae) with a description of *Monopelopia zhengi* Lin sp. n. Zootaxa.

[27] Liu J., Tang H. (2017). *Djalmabatista* Fittkau, 1968 (Diptera: Chironomidae: Tanypodinae) from Oriental China, with the description of a new species. Pan-Pac. Entomol..

[28] Kim S., Kim H., Shin S.C. (2016). Complete mitochondrial genome of the Antarctic midge Parochlus steinenii (Diptera: Chironomidae). Mitochondrial DNA A.

[29] Bolger A.M., Lohse M., Usadel B. (2014). Trimmomatic: A flexible trimmer for Illumina sequence data. Bioinformatics.

[30] Dierckxsens N., Mardulyn P., Smits G. (2017). NOVOPlasty: De novo assembly of organelle genomes from whole genome data. Nucleic Acids Res..

[31] Kearse M., Moir R., Wilson A., Stones-Havas S., Cheung M., Sturrock S., Buxton S., Cooper A., Markowitz S., Duran C. (2012). Geneious Basic: An integrated and extendable desktop software platform for the organization and analysis of sequence data. Bioinformatics.

[32] Bernt M., Donath A., Jühling F., Externbrink F., Florentz C., Fritzsch G., Pütz J., Middendorf M., Stadler P.F. (2013). MITOS: Improved de novo metazoan mitochondrial genome annotation. Mol. Phylogenet. Evol..

[33] Rozas J., Ferrer-Mata A., Sánchez-Delbarrio J.C., Guirao-Rico S., Librado P., Ramos-Onsins S.E., Sánchez-Gracia A. (2017). DnaSP 6: DNA sequence polymorphism analysis of large data sets. Mol. Biol. Evol..

[34] Edgar R.C. (2004). MUSCLE: Multiple sequence alignment with high accuracy and high throughput. Nucleic Acids Res..

[35] Capella-Gutiérrez S., Silla-Martínez J.M., Gabaldón T. (2009). trimAl: A tool for automated alignment trimming in large-scale phylogenetic analyses. Bioinformatics.

[36] Kück P., Longo G.C. (2014). FASconCAT-G: Extensive functions for multiple sequence alignment preparations concerning phylogenetic studies. Front. Zool..

[37] Xia X. (2013). DAMBE5: A comprehensive software package for data analysis in molecular biology and evolution. Mol. Biol. Evol..

[38] Kück P., Meid S.A., Groß C., Wägele J.W., Misof B. (2014). AliGROOVE–visualization of heterogeneous sequence divergence within multiple sequence alignments and detection of inflated branch support. BMC Bioinform..

[39] Lanfear R., Frandsen P.B., Wright A.M., Senfeld T., Calcott B. (2017). PartitionFinder 2: New methods for selecting partitioned models of evolution for molecular and morphological phylogenetic analyses. Mol. Biol. Evol..

[40] Minh B.Q., Schmidt H.A., Chernomor O., Schrempf D., Woodhams M.D., Von Haeseler A., Lanfear R. (2020). IQ-TREE 2: New models and efficient methods for phylogenetic inference in the genomic era. Mol. Biol. Evol..

[41] Ronquist F., Teslenko M., Van Der Mark P., Ayres D.L., Darling A., Höhna S., Larget B., Liu L., Suchard M.A., Huelsenbeck J.P. (2012). MrBayes 3.2: Efficient Bayesian phylogenetic inference and model choice across a large model space. Syst. Biol..

[42] Rambaut A., Drummond A.J., Xie D., Baele G., Suchard M.A. (2018). Posterior summarization in Bayesian phylogenetics using Tracer 1.7. Syst. Biol..

[43] Schmidt H.A., Von Haeseler A. (2007). Maximum-likelihood analysis using TREE-PUZZLE. Curr. Protoc. Bioinform..

[44] Oteo-Garcia G., Oteo J.A. (2019). Evolutionary distances corrected for purifying selection and ancestral polymorphisms. J. Theor. Biol..

[45] Johri P., Charlesworth B., Jensen J.D. (2020). Toward an evolutionarily appropriate null model: Jointly inferring demography and purifying selection. Genetics.

[46] James J.E., Piganeau G., Eyre-Walker A. (2016). The rate of adaptive evolution in animal mitochondria. Mol. Ecol..

[47] Song N., Zhang H., Zhao T. (2019). Insights into the phylogeny of Hemiptera from increased mitogenomic taxon sampling. Mol. Phylogenet. Evol..

[48] Krosch M., Cranston P., Bryant L., Strutt F., Mccluen S. (2017). Towards a dated molecular phylogeny of the *Tanypodinae* (Chironomidae, Diptera). Invertebr. Syst..

