# Supplementary material for: Comparative Mitogenomic Analyses of Tanypodinae (Diptera: Chironomidae)
Source: Insects. 2025 Feb 12;16(2):203. doi: 10.3390/insects16020203 (PMC11855973; doi:10.3390/insects16020203)
Supplement: Supplementary file 1 [file insects-16-00203-s001.zip › insects-3435203-supplementary.pdf]

Supplementary materials

Table S1. The best model for each partition of the four datasets.

Datasets	Best model
AA	mtART+R4
PCG123	GTR+F+R4
PCG123R	GTR+F+R5
PCG12	GTR+F+I+G4

Table S2. Nucleotide composition of mitochondrial genomes of 21 Tanypodinae species.

	Species	Whole Genome	Protein Coding Genes	1st Codon Position	2nd Codon Position	3rd Codon Position	tRNA Genes	12S rRNA	16S rRNA
A+T %	*Ablabesmyia monilis*	76.14	72.64	67.05	66.21	86.91	77.10	79.03	81.31
	Ablabesmyia prorasha	76.42	73.65	67.02	65.97	85.89	76.10	79.35	82.32
	Anatopynia plumipes	77.46	74.75	67.42	65.10	91.44	76.80	79.90	81.79
	Clinotanypus yani	75.37	73.77	65.42	65.07	85.65	75.70	80.34	83.41
	Conchapelopia togamaculosa	75.64	74.34	66.75	65.68	87.12	76.80	81.33	82.59
	Denopelopia bractea	76.94	74.85	68.52	66.13	89.12	76.90	80.49	82.60
	Djalmabatista sinica	77.33	72.52	67.66	65.15	89.12	77.70	79.93	83.19
	Larsia myagsensis	79.63	75.31	70.30	67.71	90.49	80.60	79.65	82.17
	Macropelopia paranebulosa	77.05	72.30	66.86	65.33	88.33	77.80	80.30	83.50
	Monopelopia zhengi	77.98	71.82	67.58	65.65	91.86	76.80	79.05	82.16
	Natarsia qinlingica	75.01	74.24	66.04	65.04	82.12	76.00	79.18	81.53
	Psectrotanypus dyari	74.82	73.21	65.93	65.17	83.61	76.80	79.01	81.34
	Procladius longistilus	77.20	72.03	67.40	64.96	91.07	75.70	80.80	82.96
	Saetheromyia tedoriprima	76.71	71.30	66.52	65.20	88.80	76.50	82.35	83.16
	Tanypus chinensis	75.81	72.99	67.48	65.12	84.59	76.20	81.85	82.73

	Tanypus kraatzi	75.77	73.77	66.52	65.23	85.07	76.60	80.17	82.74
	Tanypus punctipennis	75.62	76.43	66.01	65.04	85.51	76.10	83.87	82.86
	Thienemannimyia fuscipes	74.44	73.44	66.14	65.57	83.64	76.40	79.18	81.73
	Thienemannimyia tripunctata	76.35	74.92	66.39	65.58	86.26	76.00	81.08	85.85
	Trissopelopia sp. 1XL	78.34	72.44	69.10	66.46	90.70	78.80	78.90	80.95
	Zavrelimyia dolosa	78.06	75.69	67.21	65.60	89.41	77.40	78.39	81.56
AT-skew	*Ablabesmyia monilis*	0.02	-0.17	-0.08	-0.38	-0.11	0.02	0.00	-0.03
	Ablabesmyia prorasha	0.03	-0.18	-0.07	-0.39	-0.10	0.04	-0.03	-0.06
	Anatopynia plumipes	0.01	-0.17	-0.11	-0.39	-0.09	0.01	-0.01	-0.12
	Clinotanypus yani	0.01	-0.19	-0.10	-0.38	-0.10	0.02	-0.08	-0.07
	Conchapelopia togamaculosa	0.03	-0.18	-0.09	-0.38	-0.09	0.02	-0.05	-0.07
	Denopelopia bractea	0.01	-0.18	-0.09	-0.39	-0.09	0.02	-0.04	-0.05
	Djalmabatista sinica	0.02	-0.17	-0.10	-0.38	-0.05	0.02	-0.03	-0.07
	Larsia myagsensis	0.03	-0.19	-0.06	-0.39	-0.08	0.05	-0.07	-0.07
	Macropelopia paranebulosa	0.02	-0.18	-0.08	-0.39	-0.12	0.01	-0.06	-0.09
	Monopelopia zhengi	0.02	-0.18	-0.09	-0.38	-0.11	0.02	-0.02	-0.06
	Natarsia qinlingica	0.02	-0.17	-0.10	-0.39	-0.08	0.02	-0.02	-0.08
	Psectrotanypus dyari	0.03	-0.18	-0.09	-0.39	-0.10	0.03	-0.07	-0.07
	Procladius longistilus	0.02	-0.18	-0.11	-0.38	-0.07	0.02	-0.02	-0.05
	Saetheromyia tedoriprima	0.05	-0.18	-0.11	-0.38	-0.09	0.02	-0.05	-0.10
	Tanypus chinensis	0.04	-0.18	-0.09	-0.38	-0.07	0.03	-0.07	-0.02
	Tanypus kraatzi	0.04	-0.18	-0.09	-0.38	-0.08	0.03	-0.06	-0.06

	Tanypus punctipennis	0.04	-0.17	-0.09	-0.38	-0.09	0.04	-0.05	-0.06
	Thienemannimyia fuscipes	0.05	-0.18	-0.08	-0.38	-0.09	0.03	-0.03	-0.06
	Thienemannimyia tripunctata	0.02	-0.18	-0.10	-0.38	-0.10	0.02	-0.05	-0.07
	Trissopelopia sp. 1XL	0.02	-0.18	-0.07	-0.39	-0.08	0.03	-0.05	-0.07
	Zavrelimyia dolosa	0.01	-0.17	-0.10	-0.38	-0.09	0.02	-0.04	-0.07
GC-skew	*Ablabesmyia monilis*	-0.16	0.02	0.24	-0.16	0.01	-0.08	0.27	0.34
	Ablabesmyia prorasha	-0.17	0.03	0.24	-0.15	-0.17	-0.11	0.22	0.30
	Anatopynia plumipes	-0.13	0.05	0.30	-0.16	-0.06	-0.08	0.25	0.34
	Clinotanypus yani	-0.20	0.03	0.24	-0.18	-0.18	-0.12	0.23	0.29
	Conchapelopia togamaculosa	-0.15	0.05	0.26	-0.16	-0.11	-0.08	0.21	0.29
	Denopelopia bractea	-0.17	0.04	0.27	-0.13	-0.11	-0.12	0.30	0.30
	Djalmabatista sinica	-0.16	0.02	0.29	-0.16	-0.13	-0.09	0.25	0.32
	Larsia myagsensis	-0.17	0.05	0.24	-0.11	-0.18	-0.07	0.23	0.31
	Macropelopia paranebulosa	-0.14	0.00	0.26	-0.16	-0.03	-0.08	0.23	0.29
	Monopelopia zhengi	-0.14	0.02	0.27	-0.15	-0.08	-0.07	0.25	0.30
	Natarsia qinlingica	-0.19	0.03	0.25	-0.16	-0.19	-0.09	0.24	0.36
	Psectrotanypus dyari	-0.20	0.01	0.23	-0.15	-0.07	-0.11	0.29	0.35
	Procladius longistilus	-0.13	0.02	0.32	-0.18	-0.04	-0.09	0.25	0.29
	Saetheromyia tedoriprima	-0.16	-0.01	0.29	-0.17	-0.05	-0.09	0.23	0.32
	Tanypus chinensis	-0.21	0.04	0.28	-0.16	-0.16	-0.08	0.22	0.29
	Tanypus kraatzi	-0.18	0.04	0.26	-0.16	-0.11	-0.09	0.25	0.26
	Tanypus punctipennis	-0.17	0.03	0.28	-0.16	-0.11	-0.10	0.25	0.30

	Thienemannimyia fuscipes	-0.17	0.02	0.24	-0.16	-0.09	-0.07	0.23	0.30
	Thienemannimyia tripunctata	-0.14	0.05	0.26	-0.16	-0.03	-0.09	0.22	0.29
	Trissopelopia sp. 1XL	-0.15	0.03	0.26	-0.15	-0.12	-0.07	0.25	0.33
	Zavrelimyia dolosa	-0.17	0.03	0.28	-0.15	-0.03	-0.10	0.24	0.31

Table S3. Start and stop codons of 13 Protein Coding Genes in the mitogenomes of 21 Tanypodinae species.

Feature	Start/Stop codon												
	ATP8	*ATP6*	*CO1*	*CO2*	*CO3*	*CytB*	*ND1*	*ND2*	*ND3*	*ND4*	*ND4L*	*ND5*	*ND6*
Ablabesmyia monilis	ATT/TAA	ATG/TAA	TCG/TAA	ATG/T-	ATG/TAA	ATG/TAA	TTG/TAA	ATT/TAA	ATT/TAA	ATG/TAA	ATG/TAA	GTG/TAA	ATT/TAA
Ablabesmyia prorasha	ATT/TAA	ATG/TAA	TCG/TAA	ATG/T-	ATG/TAA	ATG/TAA	TTG/TAA	ATC/TAA	ATT/TAA	ATG/TAA	ATG/TAA	GTG/TAA	ATT/TAA
Anatopynia plumipes	ATT/TAA	ATG/TAA	TTG/TAA	ATG/T-	ATG/TAA	ATG/TAA	TTG/TAA	ATT/TAA	ATC/TAA	ATG/TAA	ATG/TAA	GTG/TAA	ATT/TAA
Clinotanypus yani	ATT/TAA	ATG/TAA	TCG/TAA	ATG/T-	ATG/TAA	ATG/TAA	TTG/TAA	ATT/TAA	ATT/TAA	ATG/TAA	ATG/TAA	GTG/TAA	ATT/TAA
Conchapelopia togamaculosa	ATT/TAA	ATG/TAA	TTG/TAA	ATG/T-	ATG/TAA	ATG/TAA	TTG/TAA	ATT/TAA	ATT/TAA	ATG/TAA	ATG/TAA	GTG/TAA	ATT/TAA
Denopelopia bractea	ATT/TAA	ATG/TAA	TTG/TAA	ATG/T-	ATG/TAA	ATG/TAA	TTG/TAA	ATT/TAA	ATT/TAA	ATG/TAA	ATG/TAA	GTG/TAA	ATT/TCT
Djalmabatista sinica	ATT/TAA	ATG/TAA	TCG/TAA	ATG/T-	ATG/TAA	ATG/TAA	TTG/TAA	ATT/TAA	ATT/TAA	ATG/TAA	ATG/TAA	GTG/TAA	ATT/TAA
Larsia myagsensis	ATT/TAA	ATG/TAA	TCG/TAA	ATG/T-	ATG/TAA	ATG/TAA	TTG/TAA	ATT/TAA	ATT/TAG	ATG/TAA	ATG/TAA	GTG/TTT	ATT/TAA
Macropelopia paranebulosa	ATT/TAA	ATG/TAA	TCG/TAA	ATG/T-	ATG/TAA	ATG/TAA	TTG/TAA	ATT/TAA	ATT/TAA	ATG/TAA	ATG/TAA	GTG/TAA	ATT/TAA
Monopelopia zhengi	ATT/TAA	ATG/TAA	TCG/TAA	ATG/T-	ATG/TAA	ATG/TAA	TTG/TAA	ATT/TAA	ATT/TAA	ATG/TAA	ATG/TAA	ATG/TAA	ATT/TAA
Natarsia qinlingica	ATT/TAA	ATG/TAA	TCG/TAA	ATG/T-	ATG/TAA	ATG/TAA	TTG/TAA	ATT/TAA	ATC/TAA	ATG/TAA	ATG/TAA	GTG/TAA	ATT/TAA
Psectrotanypus dyari	ATT/TAA	ATG/TAA	TCG/TAA	ATG/T-	ATG/TAA	ATG/TAA	TTG/TAA	ATT/TAA	ATT/TAA	ATG/TAA	ATG/TAA	ATC/TAA	ATT/TAA
Procladius longistilus	ATT/TAA	ATG/TAA	TCG/TAA	ATG/T-	ATG/TAA	ATG/TAA	TTG/TAG	ATT/TAA	ATT/TAA	ATG/TAA	ATG/TAA	GTG/TAA	ATT/TAA
Saetheromyia tedoriprima	ATT/TAA	ATG/TAA	TCG/TAA	ATG/T-	ATG/TAA	ATG/TAA	TTG/TAA	ATT/TAA	ATT/TAA	ATG/TAA	ATG/TAA	GTG/TAA	ATT/TAA
Tanypus chinensis	ATT/TAA	ATG/TAA	ACG/TAA	ATG/T-	ATG/TAA	ATG/TAA	GTG/TAG	ATT/TAG	ATT/TAA	ATG/TAA	ATG/TAA	GTG/TAA	ATT/TAA

Tanypus kraatzi	ATA/TAA	ATG/TAA	ACG/TAA	ATG/T-	ATG/TAA	ATG/TAG	ATG/TAA	ATT/TAA	ATT/TAA	ATG/TAG	ATG/TAA	GTG/TAA	ATT/TAA
Tanypus punctipennis	ATT/TAA	ATG/TAA	ACG/TAA	ATG/T-	ATG/TAA	ATG/TAA	GTG/TAA	ATT/TAA	ATT/TAA	ATG/TAG	ATG/TAA	GTG/TAA	ATT/TAA
Thienemannimyia fuscipes	ATT/TAA	ATG/TAA	TCG/TAA	ATG/T-	ATG/TAA	ATG/TAA	TTG/TAA	ATT/TAA	ATT/TAA	ATG/TAG	ATG/TAA	GTG/TAA	ATC/TAA
Thienemannimyia tripunctata	ATT/TAA	ATG/TAA	TCG/TAA	ATG/T-	ATG/TAA	ATG/TAA	TTG/TAA	ATT/TAA	ATT/TAA	ATG/TAA	ATG/TAA	ATC/TAA	ATT/TAA
Trissopelopia sp. 1XL	ATT/TAA	ATG/TAA	TCG/TAA	ATG/T-	ATG/TAA	ATG/TAA	TTG/TAA	ATT/TAA	ATT/TAA	ATG/TAA	ATG/TAA	ATC/TAA	ATT/TAA
Zavrelimyia dolosa	ATC/TAA	ATG/TAA	TCG/TAA	ATG/T-	ATG/TAA	ATG/TAA	TTG/TAA	ATT/TAA	ATT/TAA	ATG/TAA	ATG/TAA	GTG/TAA	ATT/TAA

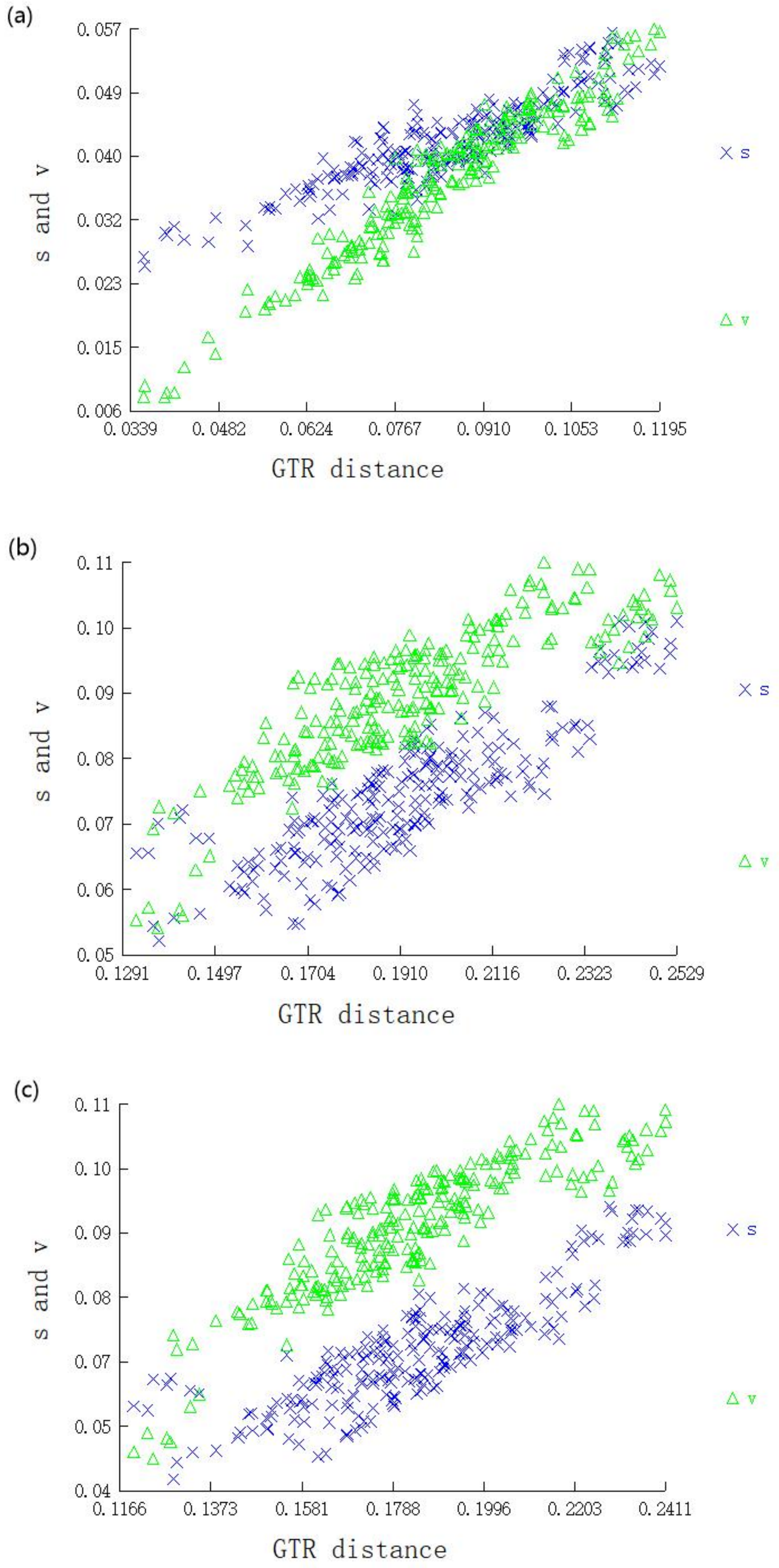

Figure S1. Substitution patterns of the PCG12 (a), PCG123 (b), and PCG123R (c) datasets. The graphs represent the increase in GTR distance.

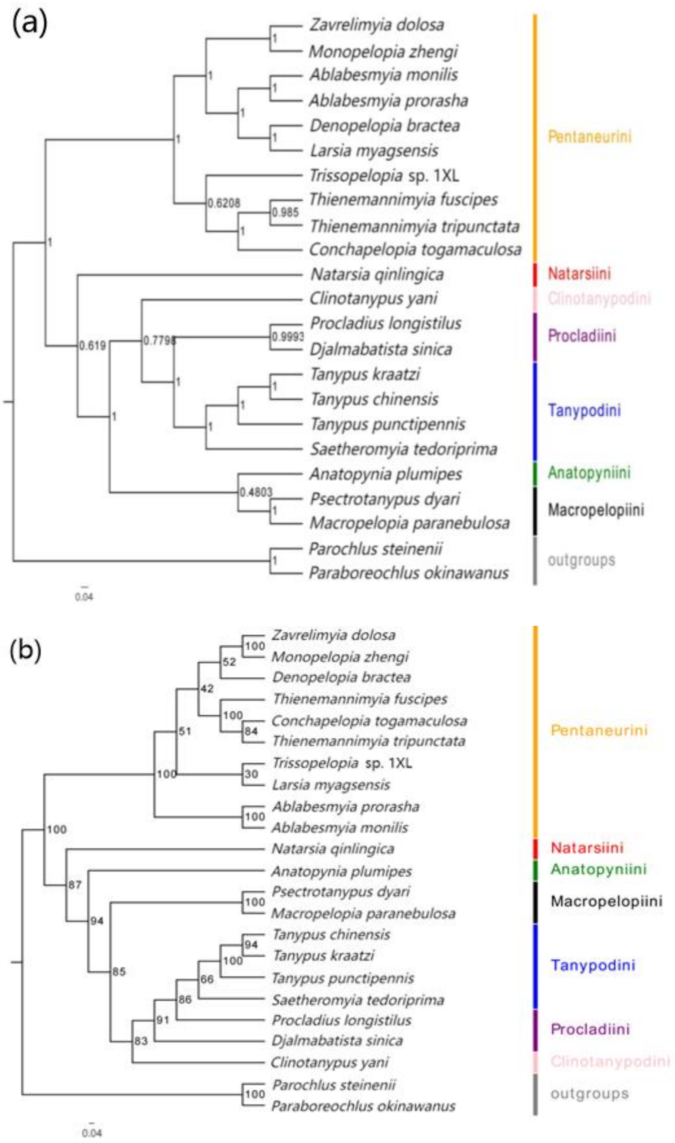

Figure S2. Phylogenetic trees of Tanypodinae inferred from the AA dataset (with *Larsia myagsensis*). (a) Bayesian Inference tree. Numbers at the nodes are BI posterior probabilities. (b) Maximum Likelihood tree. Numbers at the nodes are ML bootstrap values.

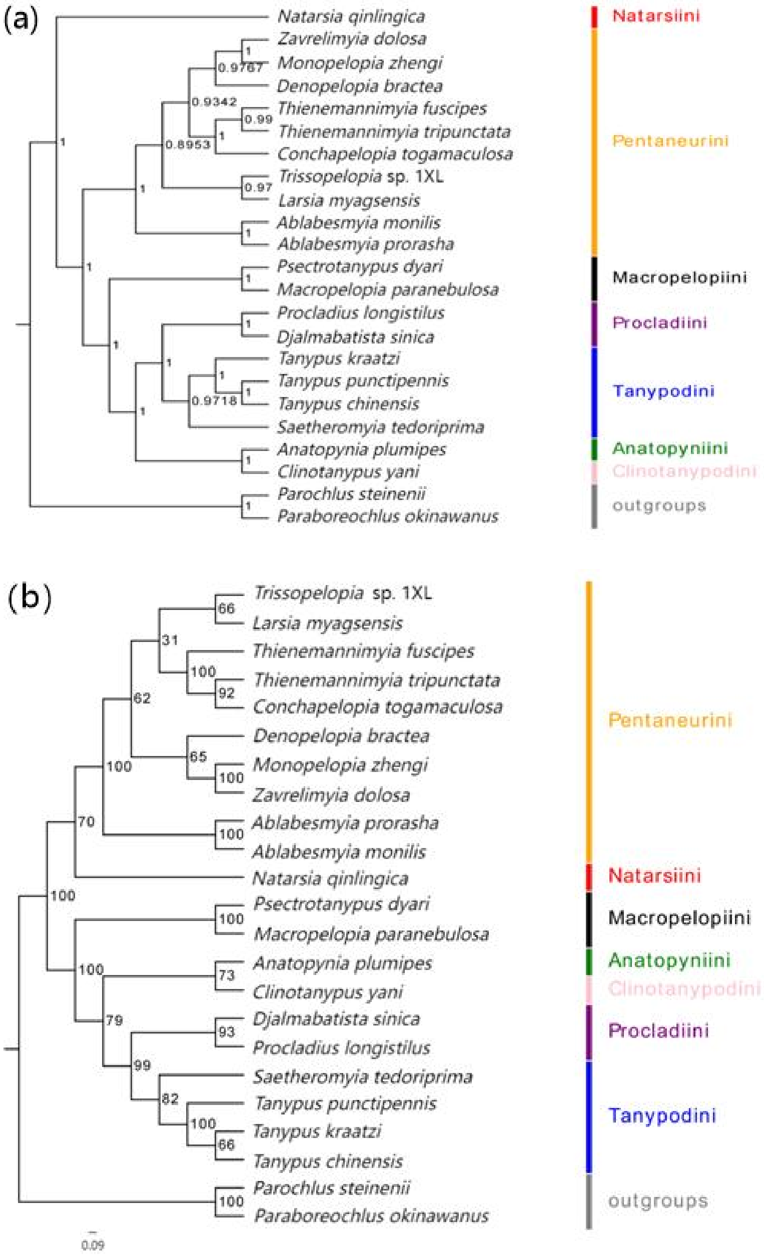

Figure S3. Phylogenetic trees of Tanypodinae inferred from the PCG123 dataset. (with *L. myagsensis*). (a) Bayesian Inference tree. Numbers at the nodes are BI posterior probabilities. (b) Maximum Likelihood tree. Numbers at the nodes are ML bootstrap values.

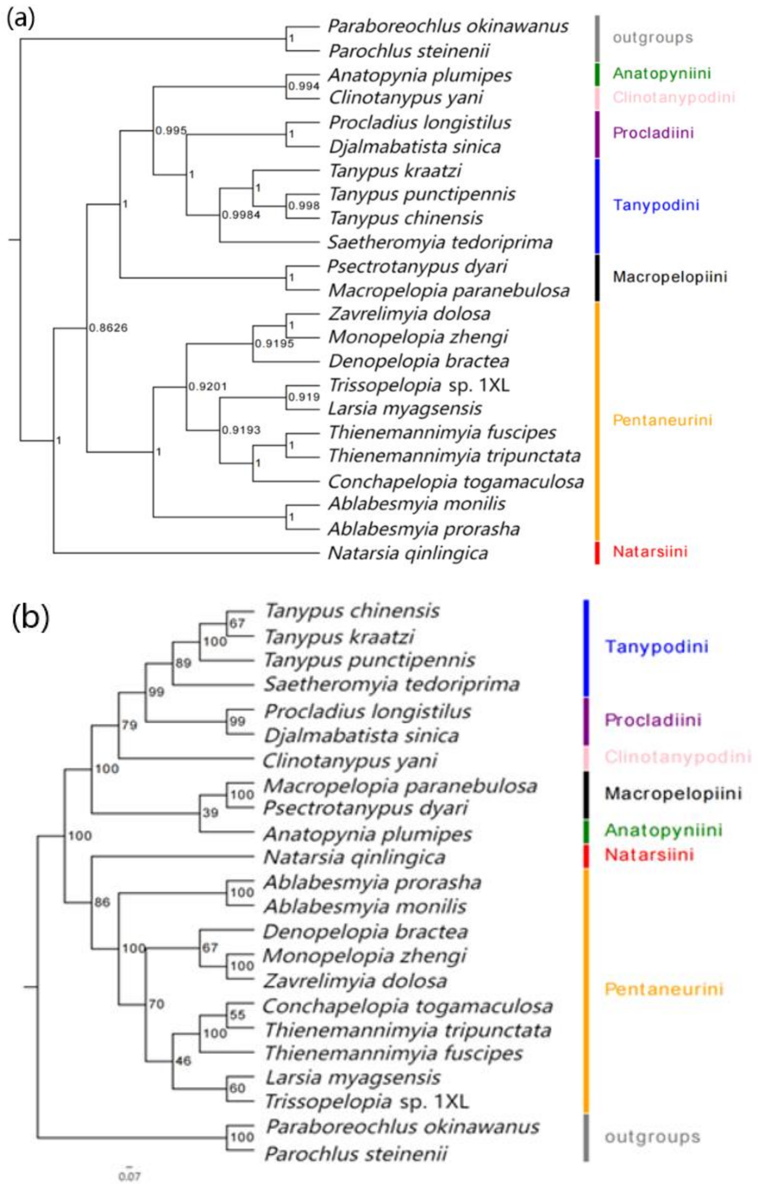

Figure S4. Phylogenetic trees of Tanypodinae inferred from the PCG123R dataset (with *L. myagsensis*). (a) Bayesian Inference tree. Numbers at the nodes are BI posterior probabilities. (b) Maximum Likelihood tree. Numbers at the nodes are ML bootstrap values.

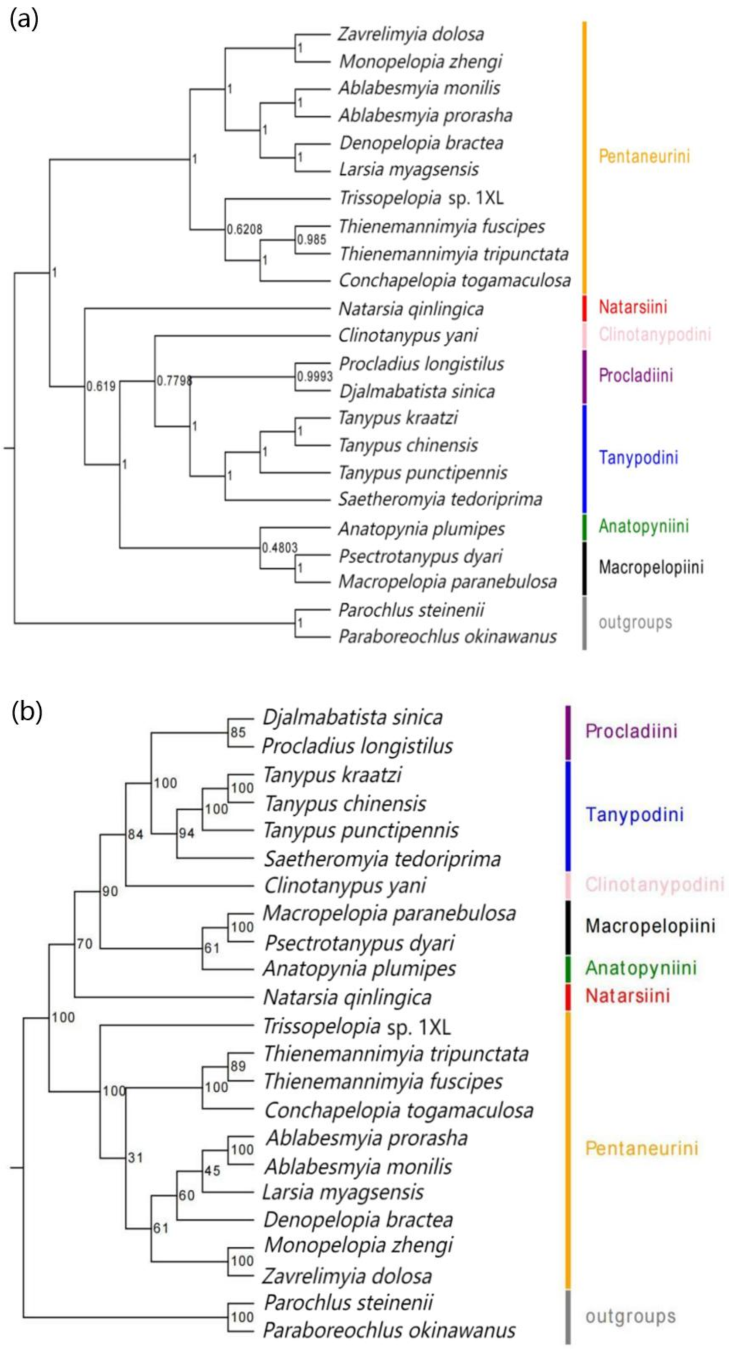

Figure S5. Phylogenetic trees of Tanypodinae inferred from the PCG12 dataset (with *L. myagsensis*). (a) Bayesian Inference tree. Numbers at the nodes are BI posterior probabilities. (b) Maximum Likelihood tree. Numbers at the nodes are ML bootstrap values.

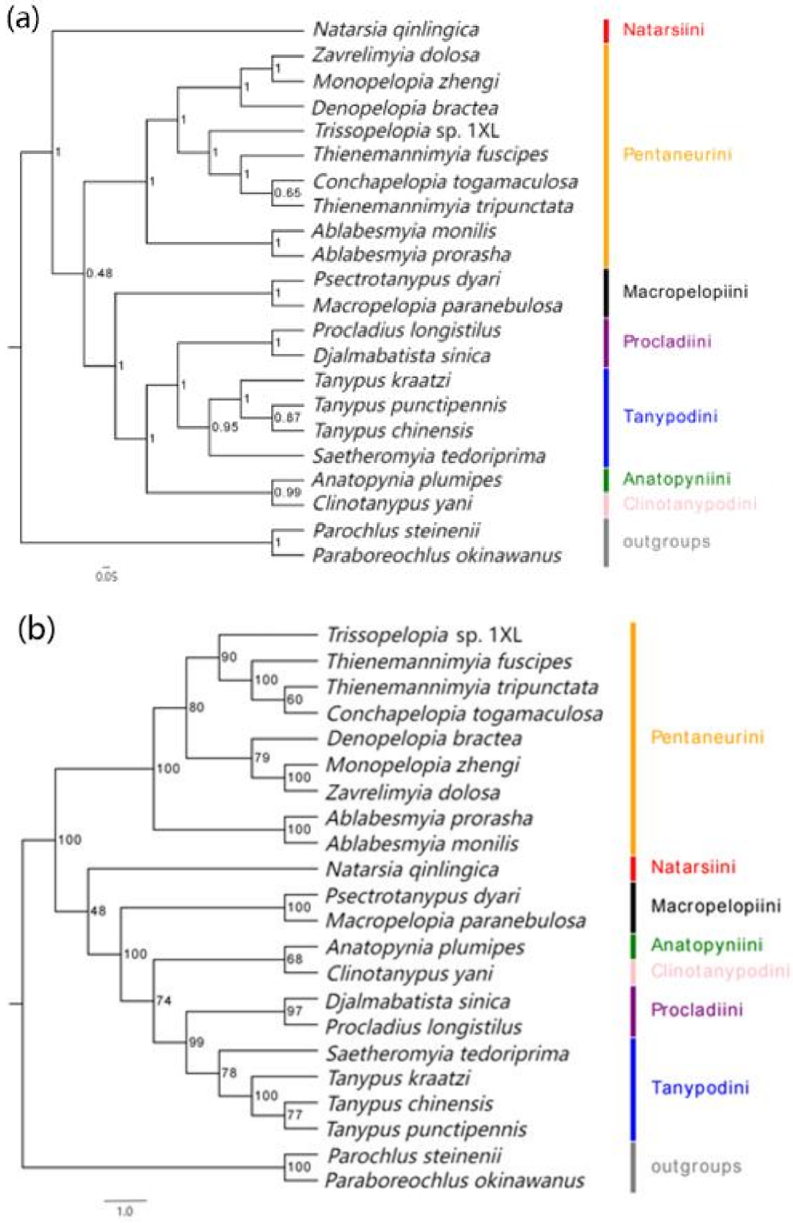

Figure S6. Phylogenetic trees of Tanypodinae inferred from the PCG123 dataset. (without *L. myagsensis*). (a) Bayesian Inference tree. Numbers at the nodes are BI posterior probabilities. (b) Maximum Likelihood tree. Numbers at the nodes are ML bootstrap values.

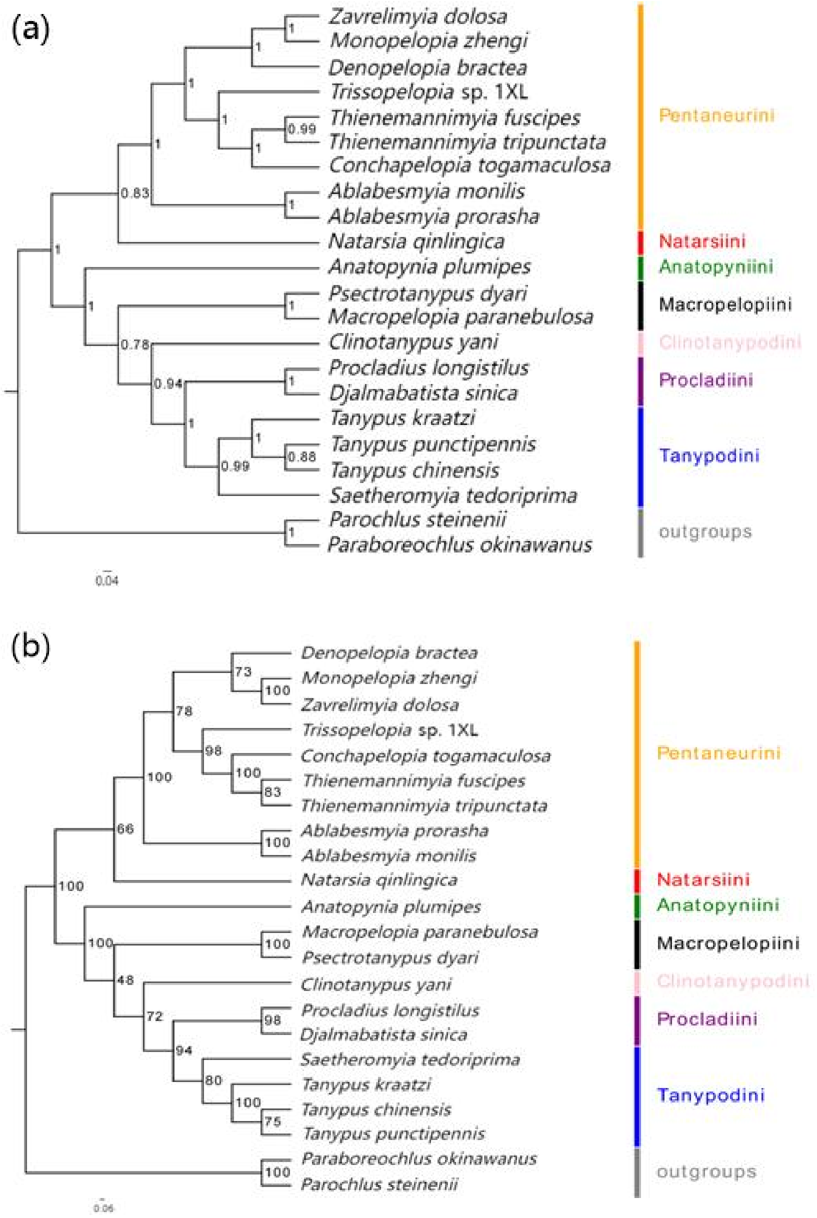

Figure S7. Phylogenetic trees of Tanypodinae inferred from the PCG123R dataset. (without *L. myagsensis*). (a) Bayesian Inference tree. Numbers at the nodes are BI posterior probabilities. (b) Maximum Likelihood tree. Numbers at the nodes are ML bootstrap values.

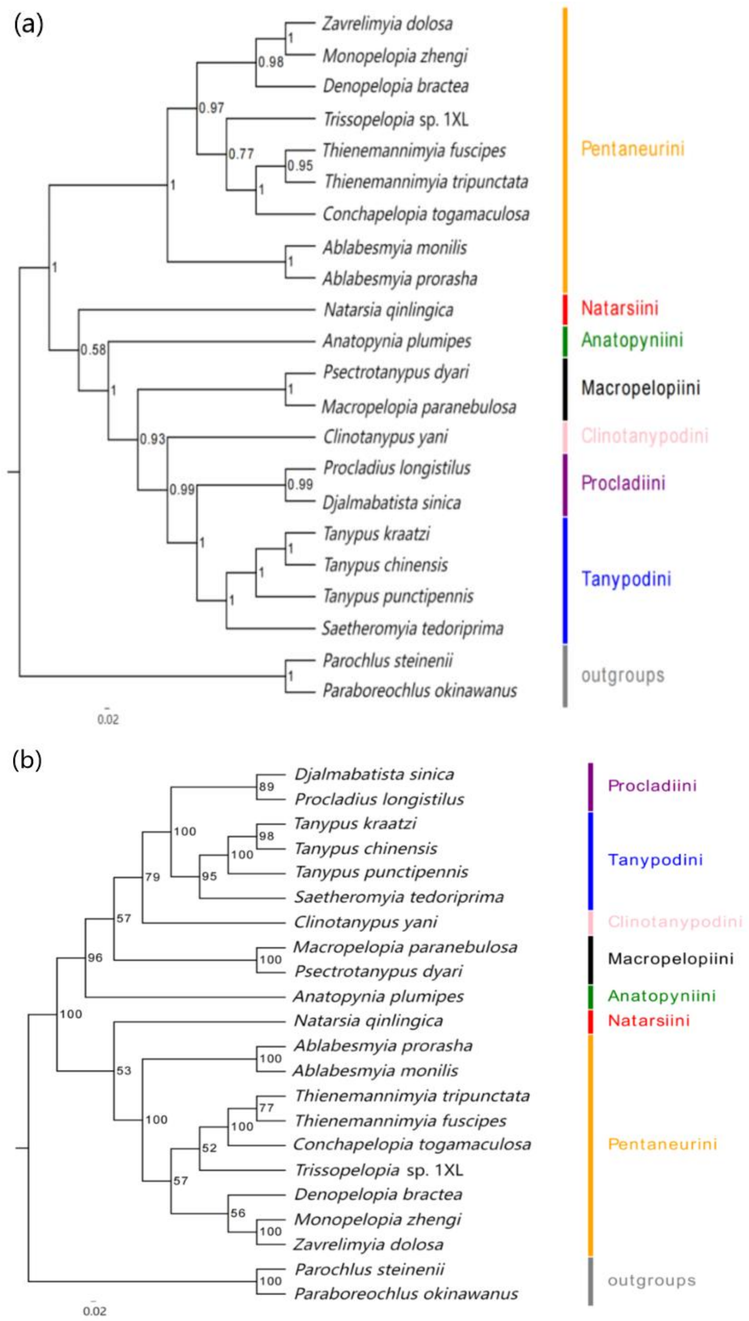

Figure S8. Phylogenetic trees of Tanypodinae inferred from the PCG12 dataset. (without *L. myagsensis*). (a) Bayesian Inference tree. Numbers at the nodes are BI posterior probabilities. (b) Maximum Likelihood tree. Numbers at the nodes are ML bootstrap values.
